# Validation and standardization of the Childhood Trauma Screener among Romanian children, adolescents, and college students

**DOI:** 10.1002/jts.70038

**Published:** 2026-01-09

**Authors:** Oana Alexandra David, Cristina Teodora Tomoiagă, Ioana Alexandra Iuga

**Affiliations:** ^1^ Department of Clinical Psychology and Psychotherapy Babeş‐Bolyai University Cluj‐Napoca Romania; ^2^ DATA Lab, The International Institute for the Advanced Studies of Psychotherapy and Applied Mental Health Babes‐Bolyai University Cluj‐Napoca Romania; ^3^ Evidence‐Based Psychological Assessment and Interventions Doctoral School Babeș‐Bolyai University Cluj‐Napoca Romania

## Abstract

Childhood trauma, defined as violence against individuals under 18 years of age, affects 1,000,000,000 children globally, with neglect being the most common form. Such trauma is linked to various mental health issues and poor emotional regulation. This study aimed to validate the Childhood Trauma Screener (CTS) in a sample of Romanian children, adolescents, and college students, utilizing data from two separate samples: 1,221 first‐year college students and 216 children and adolescents aged 8–16 years. We assessed validity using bivariate correlations, reliability using Cronbach's alpha (.63–.75), and structure using confirmatory factor analyses. In the college student sample, higher childhood trauma scores were associated with lower self‐compassion, *r*s = −.24–−.29, *p* < .001, and reappraisal use, *r*s = −.10–−.22, *p* < .001; higher suppression use for exposure to neglect, *r* = .16, *p* < .001, and overall trauma, *r* = −.11, *p* < .001; and more mental health difficulties, *r*s = .29–.31, *p* < .001. In the child and adolescent sample, higher childhood trauma scores were associated with increased emotional and behavioral difficulties, *rs* = .28–.45, *p* < .001, and poorer emotion regulation abilities, particularly lower emotion control and reduced emotional self‐awareness, *r*s = .16–.43, *p* < .001. The results from both samples supported a two‐factor model of abuse and neglect.

The World Health Organization (WHO) defines childhood trauma as abuse and neglect perpetrated against people under 18 years old (WHO, 2024). Globally, 1,000,000,000 children and adolescents aged 2–17 years have experienced one such form of maltreatment, making childhood trauma an important concern and highly prioritized global issue. Data from the National Children's Alliance (2022) shows that neglect constitutes the most prevalent form of trauma, accounting for 76% of cases, followed by physical abuse (16%), sexual abuse (10%), and sex trafficking (0.2%).

All forms of childhood trauma are considered risk factors for the later onset of various mental health concerns (Baldwin et al., 2023; Kovačević et al., 2022; Rosado et al., 2021), poor emotion regulation (Hoffman et al., 2023; Yin et al., 2022), and lower levels of self‐compassion (Zhang et al., 2023). Moreover, a WHO report indicates that individuals who experience abuse are more likely to exhibit aggressive behavior toward others, potentially perpetuating a cycle of abuse that affects an increasing number of people (WHO, 2024). Given the significance of childhood trauma, trauma‐focused interventions have been developed and validated (e.g., Cohen et al., 2012; David & Fodor, 2023a, 2023b). These interventions typically aim to address the traumatic experience and/or help individuals develop important resilience skills to reduce the event's impact on their mental health.

Trauma assessment is essential for providing necessary posttrauma support and interventions. Given the high prevalence of mental health disorders in children, adolescents, and adults (Miljkovic et al., 2024), it is essential to evaluate past traumatic experiences in this population and consider these experiences within a personalized intervention framework. Thus, the aim of this study was to validate the Childhood Trauma Screener (CTS; Witt et al., 2022) among children, adolescents, and college students from Romania. The CTS is a brief, five‐item self‐report questionnaire designed to assess core dimensions of childhood trauma, including sexual, emotional, and physical abuse and emotional and physical neglect in both youth and adult samples. Derived from the German version of the 28‐item Childhood Trauma Questionnaire (CTQ; Bernstein et al., 1997), the CTS has demonstrated strong correlations with corresponding CTQ dimensions and a high correlation with the total CTQ score (Grabe et al., 2012). The present study aimed to validate this brief screening tool in two distinct samples: first‐year college students (i.e., *college student sample*) children and adolescents (i.e., *child/adolescent sample*).

## METHOD

### Participants and procedure

The first sample comprised 1,221 first‐year Romanian college students (77.6% female, age range: 18–46 years, *M*_age_ = 19.38 years, *SD* = 2.10) from the largest university in Cluj‐Napoca, Romania who participated in a study on mental health among college students. Data were collected online through a platform distributed via professors, student organizations, and email invitations. Participation was voluntary, and students provided informed consent before completing the questionnaire. The second sample comprised 216 children and adolescents (55.6% female, age range: 8–16 years, *M*_age_ = 11.10 years, *SD* = 1.53). At the start of the testing session, a psychologist explained the study's purpose, guided participants through the assessment, assisted with the platform log‐in (*RE*Think), and ensured proper questionnaire completion. Parents provided informed consent for their children's participation. All procedures performed were in accordance with the ethical standards of the institutional research committee, and all participants provided informed consent prior to the beginning of the study. The study was approved by the Scientific Council of the Babeș‐Bolyai University (14.182/14.10.2022, 14.533/ 12.10.2023) and preregistered on ClinicalTrials.gov (NCT05653908, NCT06085391).

### Measures

#### Childhood trauma

The CTS (Witt et al., 2022) was used to evaluate past trauma among all participants. This five‐item scale assesses physical, emotional, and sexual and physical and emotional neglect experiences, with each item rated on a 5‐point Likert scale ranging from 1 (*never true*) to 5 (*very often true*). The scale has demonstrated adequate psychometric properties in samples of children (David & Fodor, 2023a, 2023b) and adults (Witt et al., 2022). In both of our samples, the CTS demonstrated adequate internal consistency, with Cronbach's alpha values of .74 for both the college student sample and the child/adolescent sample. When evaluating the alternative factor structure, which includes two factors, Abuse and Neglect, internal consistency was adequate for the Abuse subscale, Cronbach's α = .70, and poor for the Neglect subscale, Cronbach's α = .63, in the college student sample. Similarly, in the child/adolescent sample, internal consistency was adequate for the Abuse subscale, Cronbach's α = .75, and poor for the Neglect subscale, Cronbach's α = .66.

#### College student sample

##### Overall mental health

In the college student sample, the 12‐item General Health Questionnaire (GHQ‐12; Goldberg & Williams, 1988) was used to evaluate the severity of mental health concerns over the past few weeks. Items are rated on a scale of 0–3, with higher scores indicating higher levels of mental health–related distress. The GHQ‐12 is a commonly utilized screening tool for psychological distress and general mental health problems. The measure has demonstrated good psychometric properties in previous studies (e.g., Laranjeira, 2008). In the current college student sample, internal consistency in the college student sample was good, Cronbach's α = .90.

##### Emotion regulation

Participants in the college student sample completed the Emotion Regulation Questionnaire (ERQ; Gross & John, 2003), a 10‐item assessment that includes four questions related to expressive suppression and six items related to cognitive reappraisal, two distinct emotion regulation strategies. Items are scored on a 7‐point Likert ranging from 1 (*strongly disagree*) to 7 (*strongly agree*), with higher scores indicating more use of a given emotion regulation strategy. The ERQ has demonstrated good psychometric properties and internal consistency in other studies (e.g., Preece et al., 2019). In the current college student sample, internal consistency was good for the Cognitive Reappraisal subscale, Cronbach's α = .88, and adequate for the Expressive Suppression subscale, Cronbach's α = .77.

##### Self‐compassion

Participants in the college student sample completed the Self‐Compassion Survey–Short‐Form (SCS‐SF; Raes et al., 2011) to assess self‐compassion. This 12‐item measure contains three subscales: Mindfulness versus Overidentification, Common Humanity versus Isolation, and Self‐Kindness versus Self‐Judgment. Responses are scored on a 5‐point Likert scale ranging from 1 (*nearly never*) to 5 (*almost always*). Scores are averaged, with negative items (e.g., self‐judgement) reverse‐coded, for a possible range of 1–5; higher scores indicate higher levels of self‐compassion. The SCS‐SF has demonstrated solid psychometric properties and internal consistency in previous studies (e.g., Kotera & Sheffield, 2020). In the current college student sample, the total scale demonstrated good internal consistency, Cronbach's α = .84.

#### Child/adolescent sample

##### Emotion regulation

The 16‐item Emotion Regulation Index for Children and Adolescents (ERICA; MacDermott et al., 2010) questionnaire was used to assess emotion regulation abilities in the child/adolescent sample. Items are scored on a 5‐point Likert scale ranging from 0 (*strong disagreement*) to 5 (*strong agreement*). The ERICA includes three subscales: Emotional Self‐Awareness, Emotional Control, and Emotional Responsiveness. The scale has demonstrated good psychometric properties in previous studies (e.g., Hughes et al., 2011). In the current child/adolescent sample, The Emotional Self‐Awareness subscale demonstrated adequate internal consistency, Cronbach's α = .74; the Emotional Control subscale demonstrated good internal consistency, Cronbach's α = .87; and the Emotional Responsiveness subscale demonstrated poor internal consistency, Cronbach's α = .60. Due to this low internal consistency, we excluded the Emotional Responsiveness subscale from all analyses.

##### Emotional and behavioral problems

The 25‐item Strengths and Difficulties Questionnaire (SDQ; Goodman, 1997) was used to assess emotional and behavioral problems and strengths in the child/adolescent sample. Along with a total score, five subscale scores can be calculated: Emotional Symptoms, Peer Relationship Problems, Conduct Problems, Prosocial Behaviors, and Hyperactivity. Items are rated on a 3‐point Likert scale ranging from 0 (*not true*) to 2 (*certainly true*). The scale has demonstrated adequate psychometric properties in previous studies (e.g., David et al., 2019). In the current child/adolescent sample, internal consistency for the total score was good, Cronbach's α = .85.

### Data analysis

All analyses were conducted using SPSS (Version 22) and JASP (Version 0.18.3.0). To assess the validity of the CTS, a series of bivariate correlation analyses was conducted to examine the associations between the scale and related constructs. A reliability analysis was conducted to determine the internal consistency of the scale, using an alpha model. Finally, the structure of the scale was analyzed for each of the samples using confirmatory factor analysis (CFA). Model fit was evaluated using several indices, including chi‐square tests and the root mean square error of approximation (RMSEA), comparative fit index (CFI), Tucker–Lewis Index (TLI), Akaike information criterion (AIC), and Bayesian information criterion (BIC). The criteria for an adequate model fit were an RMSEA value of .08 or less, a CFI value of .90 or higher, and a TLI value of .90 or higher. The analyses for the two samples were run separately.

## RESULTS

### Adult sample

#### Factor structure

To confirm the structure of the CTS, we tested the proposed one‐factor model. Although the item loadings were appropriate for this model (see Supplementary Material 1, Table S1), the fit measures indicated a poor fit (Table 1, Model 1). This result led us to consider a previously investigated two‐factor model (Witt et al., 2022). For the two‐factor model, the item loadings improved. The loadings for both the abuse (.64, .95, .41) and neglect factors (.81, .55) were positive and significant, *p*s < .001. Item 2 (emotional neglect) and Item 4 (emotional abuse) showed high factor loadings, whereas the rest of the items showed improved but moderate loadings. The two factors were moderately correlated, *r* = .64, *p* < .001. The two‐factor model demonstrated a substantially better fit than the one‐factor model (see Table 1), with a BIC change of 118, indicating very strong evidence in favor of the two‐factor solution (Raftery, 1995).

**TABLE 1:** Fit measures for the confirmatory factor analyses

Model	χ^2^	*df*	*p*	CFI	TLI	RMSEA	RMSEA 90% CI	SRMR	BIC	ΔBIC
1^a^	191	5	< .001	.87	.73	.17	[.154, .196]	.060	15,668	118
2^a^	65.9	4	< .001	.96	.89	.11	[.089, .137]	.030	15,550	
3^b^	42.0	5	< .001	.84	.69	.19	[.138, .241]	.076	3,173	32
4^b^	5.23	4	.264	.99	.99	.04	[.00, .115]	.022	3,141	

*Note*: Models 1 and 2: *N* = 1,221, Models 3 and 4: *N* = 216. *df* = degrees of freedom; CFI = comparative fit index; TLI = Tucker–Lewis Index; RMSEA = root mean square error of approximation; CI = confidence interval; SRMR = standardized root mean square residual; BIC = Bayesian information criterion.

#### Item characteristics

Table 2 presents data on the prevalence of various forms of childhood trauma assessed by the CTS, categorized by the frequency with which respondents reported experiencing these forms of abuse or neglect. The data are summarized in terms of the number of respondents and the corresponding percentage of the sample, along with cumulative percentages.

**TABLE 2:** Frequency of item‐specific Childhood Trauma Screener responses for the college sample

	1			2			3			4			5				
	(*Never true*)			(*Rarely true*)			(*Sometimes true*)			(*Often true*)			(*Very often true*)		
Item	*n*	%	Σ%	*n*	%	Σ%	*n*	%	Σ%	*n*	%	Σ%	*n*	%	Σ%	*M*	*SD*
I felt loved.	504	41.2	41.2	350	28.6	69.8	256	20.9	90.7	87	7.1	97.8	27	2.2	100.0	2.01	1.05
There was someone to take me to the doctor when I needed it.	805	65.8	65.8	230	18.8	84.6	123	10.0	94.6	50	4.1	98.7	16	1.3	100.0	1.56	0.92
People in my family hit me so hard, it left me with bruises or marks.	973	79.5	79.5	117	9.5	89.1	75	6.1	95.2	36	2.9	98.1	23	1.9	100.0	1.38	0.87
I felt that somebody in my family hated me.	759	62.0	62.0	184	15.0	77.0	122	10.0	87.0	78	6.4	93.4	81	6.6	100.0	1.81	1.24
Somebody molested me.	1,040	85.0	85.0	76	6.2	91.2	71	5.8	97.0	24	2.0	98.9	13	1.1	100.0	1.28	0.75

*Note*: Σ% = the cumulative percentage; please note that responses for items one and two are reversed.

#### Associations with variables of interest

A Pearson correlation analysis was conducted to examine the associations among childhood trauma, self‐compassion, emotion regulation, and general mental health problems. The results (see Supplementary Material 1, Table S3) indicate significant associations between multiple constructs. Overall, the associations were in the expected direction, indicating negative correlations between the CTS subscales and self‐compassion and reappraisal; this suggests that higher levels of childhood trauma were associated with lower levels of self‐compassion and the use of the reappraisal emotion regulation strategy. Moreover, positive associations were found between CTS scores and both mental health difficulties and suppression, indicating that childhood trauma was positively related to mental health difficulties and the use of maladaptive emotion regulation strategies, such as suppression, in this sample.

### Child/adolescent sample

#### Factor structure

For the child/adolescent sample, the one‐factor model also demonstrated acceptable item loadings (see Supplementary Material, Table S2) but poor fit indices (Table 1, Model 3), leading us to consider the previously described two‐factor model. In this model, the two factors—Abuse and Neglect—were moderately correlated, *r* = .51, *p* < .001. The loadings for the Abuse factor (.72, .84, .88) and the Neglect factor (.80, .73) were all positive and significant, *p*s < .001, and the fit indices were much improved (Table 1, Model 4), which suggests an excellent fit. The BIC change was 32, providing strong evidence in favor of the two‐factor structure.

#### Item characteristics for the child/adolescent sample

Similar to the results for the college student sample, the results for the child and adolescent sample indicated that a substantial majority of respondents reported never experiencing any of the forms of childhood trauma listed, particularly physical abuse and sexual abuse. Although most participants in the child/adolescent sample reported no emotional neglect or emotional abuse, higher percentages respondents indicated that they experienced these forms of trauma sometimes, often, or very often compared to the adult sample (see Table 3).

**TABLE 3:** Frequency of item‐specific Childhood Trauma Screener responses for the child and adolescent sample

Item	1			2			3			4			5				
	(*Never true*)			(*Rarely true*)			(*Sometimes true*)			(*Often true*)			(*Very often true*)		
Item	*n*	%	Σ%	*n*	%	Σ%	*n*	%	Σ%	*n*	%	Σ%	*n*	%	Σ%	*M*	*SD*
I felt loved.	110	50.9	50.9	51	23.6	74.5	34	15.7	90.3	11	5.1	95.4	10	4.6	100.0	1.89	1.13
There was someone to take me to the doctor when I needed it.	140	64.8	64.8	41	19.0	83.8	19	8.8	92.6	7	3.2	95.8	9	4.2	100.0	1.63	1.05
People in my family hit me so hard, it left me with bruises or marks.	164	75.9	75.9	16	7.4	83.3	22	10.2	93.5	8	3.7	97.2	6	2.8	100.0	1.50	1.01
I felt that somebody in my family hated me.	129	59.7	59.7	25	11.6	71.3	33	15.3	86.6	12	5.6	92.1	17	7.9	100.0	1.90	1.30
Somebody molested me.	162	75.0	75.0	17	7.9	82.9	16	7.4	90.3	10	4.6	94.9	11	5.1	100.0	1.57	1.13

*Note*: Σ% = the cumulative percentage; please note that responses for items one and two are reversed.

#### Associations with variables of interest

Associations between CTS scores and emotional and behavioral difficulties revealed that higher trauma scores were related to higher levels of psychological distress. Specifically, higher trauma scores were related to SDQ total scores (i.e., total difficulties). Additionally, adaptive emotion regulation strategies were negatively correlated with trauma variables. See Supplementary Table S4 for all correlations in the child/adolescent sample. Details regarding the cut‐off scores are provided in the Supplementary Material 2, Table S5.

## DISCUSSION

The aim of this study was to validate the Romanian version of the CTS. The validation process was conducted using two distinct samples: college students and children/adolescents. We applied the same procedure to both samples, beginning with a CFA as the first step. For both samples, the results indicated that a two‐factor model of abuse and neglect provided a better fit than a one‐factor solution, with all items significantly contributing to their respective dimensions across age groups. In the college student sample, the model demonstrated stronger stability, with smaller standard errors and a clear distinction between abuse and neglect. In the child/adolescent sample, the factor loadings were generally higher, but standard errors were also larger, indicating greater measurement variability.

Regarding the frequency of the adverse experiences, analyses conducted in the college sample showed that severe abuse experiences were less frequently reported compared to the child and adolescent sample. However, emotional neglect had higher rates of mild‐to‐moderate exposure, suggesting it is more commonly experienced. Although physical and sexual abuse were reported less frequently, participants who reported these trauma types tended to endorse more severe levels. These findings highlight a higher prevalence of emotional trauma and lower prevalence but higher severity of physical and sexual abuse in the college student sample. The results of the analysis for the child/adolescent sample showed a similar trend wherein severe abuse experiences were less frequently reported. Emotional neglect demonstrated a higher rate of mild‐to‐moderate exposure, similar to the college sample, suggesting it is more common.

Our findings indicate that higher levels of childhood trauma exposure are associated with more severe emotional and behavioral difficulties in children and adolescents. Higher levels of abuse, neglect, and overall trauma were found to be associated with higher levels of emotional and behavioral difficulties and lower levels of emotional control and emotional awareness. The literature supports these results, including evidence of an association between childhood trauma and the use of maladaptive emotion regulation strategies in a sample of adolescents and young adults (Doba et al., 2022).

In the college student sample, our findings indicate that higher degrees of childhood trauma exposure were associated with lower self‐compassion and reappraisal use and higher levels of suppression use and general mental health problems. The results were similar for higher levels of neglect. Although previous studies have demonstrated associations between past trauma exposure and the use of maladaptive emotion regulation strategies (Doba et al., 2022; Peng et al., 2021), we found that higher levels of abuse negatively and significantly predicted lower levels of reappraisal but did not observe a significant association between abuse and suppression use.

Regarding study limitations, we note the smaller sample size for the child/adolescent sample compared to the college student sample. Additionally, we did not compare the CTS to a similar instrument to assess concurrent validity. Our reliance on self‐reported data may have introduced bias; the cross‐sectional design limits causal inferences; and the sample may not be fully representative of the broader population, potentially affecting the generalizability of the findings. Moreover, our results may be biased due to the predominantly urban school samples, and the findings based on college students may not be fully representative of all youth within the same age group.

The findings have several implications. Having valid measurement tools for assessing traumatic experiences in populations of children, adolescents, and college students allows for the identification of individuals with a history of trauma and the personalization of interventions that may help mitigate long‐term psychological consequences. Valid measurements foster better understanding and support for young people affected by trauma, promoting healthier development and overall well‐being. Reliable measurements also support longitudinal studies, providing insights into the long‐term effects of trauma and informing theories of resilience and recovery. In conclusion, we found that the CTS is a valid measurement for the assessment of past traumatic experiences in children, adolescents, and college students from Romania, with high internal consistency, appropriate factor structure, and predictive validity.

## AUTHOR NOTE

This work was supported by two grants from the Romanian National Authority for Scientific Research, CNCS—UEFISCDI (PN‐III‐P4‐ID‐PCE‐2020‐2170, PN‐III‐P2‐2.1‐PED‐2021‐3882)

## OPEN PRACTICES STATEMENT

The study was preregistered at ClinicalTrials.gov (NCT05653908, NCT06085391).

The datasets generated during and/or analyzed during the current study are not publicly available but are available from the corresponding author on reasonable request at oanadavid@psychology.ro.

## AUTHOR CONTRIBUTIONS

Oana Alexandra David: conceptualization, funding acquisition, methodology, writing ‐ review and editing, project administration, supervision, resources. Cristina Teodora Tomoiagă: writing ‐ original draft, methodology, writing ‐ review and editing. Ioana Alexandra Iuga: investigation, writing ‐ original draft, methodology, writing ‐ review and editing, software, formal analysis, data curation.

## Supporting information



SUPPORTING INFORMATION

SUPPORTING INFORMATION
