# Supplementary material for: Validation and standardization of the Childhood Trauma Screener among Romanian children, adolescents, and college students
Source: J Trauma Stress. 2026 Jan 9;39(2):330–7. doi: 10.1002/jts.70038 (PMC13044384; doi:10.1002/jts.70038)
Supplement: Supplementary file 1 — SUPPORTING INFORMATION [file JTS-39-330-s002.docx]

**Supplementary Material: Supporting Tables for Study Findings**

**Table 1**

*Factor loadings, Standard Errors (SE), z-values, and p-values for CTS Indicators in the* college student *sample for the one-factor solution*

| Factor | Indicator | *r* | *SE* | *z* | *p* |
| --- | --- | --- | --- | --- | --- |
| Childhood Trauma | CTS1 | .42 | .029 | 14.4 | < .001 |
|  | CTS2 | .58 | .032 | 17.9 | < .001 |
|  | CTS3 | .62 | .026 | 24.0 | < .001 |
|  | CTS4 | .95 | .036 | 26.5 | < .001 |
|  | CTS5 | .40 | .023 | 17.3 | < .001 |

*Note*. CTS = Childhood Trauma Screener, Indicator = Item number, *r* = The correlation coefficient, *SE* = Standard Error, *z* = z-value, *p* = p-value indicating statistical significance.

**Table 2**

*Factor loadings, Standard Errors (SE), z-values, and p-values for CTS Indicators in the child and adolescent sample for the one-factor solution*

| Factor | Indicator | *r* | *SE* | *z* | *p* |
| --- | --- | --- | --- | --- | --- |
| Childhood Trauma | CTS1 | .44 | .087 | 5.12 | < .001 |
|  | CTS2 | .46 | .079 | 5.86 | < .001 |
|  | CTS3 | .71 | .069 | 10.41 | < .001 |
|  | CTS4 | .85 | .090 | 9.46 | < .001 |
|  | CTS5 | .86 | .077 | 11.10 | < .001 |

*Note*. CTS = Childhood Trauma Screener, Indicator = Item number, *r* = The correlation coefficient, *SE* = Standard Error, *z* = z-values, *p* = p-value indicating statistical significance.

**Table 3**

*Correlation analysis results for the* college student *sample*

| Variable | 1 | 2 | 3 |
| --- | --- | --- | --- |
| CTS_Neglect | - |  |  |
| CTS_Abuse | .45** | - |  |
| CTS_TOTAL | .78** | .90** | - |
| SCS_Total | -.25** | -.24** | -.29** |
| ERQ_Reapp | -.22** | -.10** | -.18** |
| ERQ_Suppr | .16** | .05 | .11** |
| GHQ_TOTAL | .31** | .29** | .35** |

*Note.* ** Correlation is significant at the 0.01 level (2-tailed).

**Table 4**

*Correlation analysis results for the child and adolescent sample*

| Variable | 1 | 2 | 3 |
| --- | --- | --- | --- |
| CTS_Abuse | - |  |  |
| CTS_Neglect | .37** | - |  |
| CTS_TOTAL | .90** | .75** | - |
| SDQ_TOTAL | .45** | .28** | .45** |
| ERICA_EM_CTRL | -.28** | -.16** | -.28** |
| ERICA_SELF_AWARE | -.36** | -.35** | -.43** |

*Note.* ** Correlation is significant at the 0.01 level (2-tailed).
