# Supplementary material for: Validation and standardization of the Childhood Trauma Screener among Romanian children, adolescents, and college students
Source: J Trauma Stress. 2026 Jan 9;39(2):330–7. doi: 10.1002/jts.70038 (PMC13044384; doi:10.1002/jts.70038)
Supplement: Supplementary file 2 — SUPPORTING INFORMATION [file JTS-39-330-s001.docx]

**Supplementary Material 2: K-Means Cluster Analysis for Determining Cutoffs Across Two Samples**

In order to distinguish different groups within the data, the cut-off of the scale was determined using the K-Means Cluster Analysis. This allowed for a data-driven method to establish thresholds that distinguish between different levels of the measured construct. The K-means cluster analyses were conducted with a specified two-cluster solution for the CTS scores in both samples. In the university sample, clusters reflected individuals with low trauma levels (*n* = 980) and high trauma levels (*n* = 244), with a cutoff score of > 10 based on the midpoint between cluster centroids. In the pre-university sample, clusters similarly reflected low trauma (*n* = 176) and high trauma (*n* = 40), with a cutoff score of > 11. ANOVA results indicated significant differences between clusters in both samples, *F*(1,1222) = 2912.5, *p* < .001 for the university sample and *F*(1,214) = 511.49, *p* < .001 for the pre-university sample (see Table 1 for cluster centroids).

**Table 1**

*Final cluster centers for the* college student *sample*

|  | Cluster | |
| --- | --- | --- |
|  | 1 | 2 |
| College student sample | 6.60 | 13.79 |
| Child and adolescent sample | 6.92 | 15.40 |
